# Firearm injuries and death: A United States epidemic with public health solutions

**DOI:** 10.1371/journal.pgph.0001913

**Published:** 2023-05-24

**Authors:** Kristen L. Mueller, Nakita N. Lovelady, Megan L. Ranney

**Affiliations:** 1 Department of Emergency Medicine, Washington University in St. Louis School of Medicine, St. Louis, MO, United States of America; 2 Department of Health Behavior and Health Education, College of Public Health, University of Arkansas for Medical Sciences, Little Rock, AR, United States of America; 3 Department of Emergency Medicine, Alpert Medical School, Brown University, Providence RI, United States of America; 4 Department of Behavioral and Social Science, School of Public Health, Brown University, Providence, RI, United States of America; PLOS: Public Library of Science, UNITED STATES

Firearm injuries and death are an eminently preventable public health epidemic in the United States (U.S.). Globally, for the last several decades, U.S. firearm homicide rates far outpaced those of other high-income countries, with more than 45,000 people killed by firearms in the U.S. in 2020 alone–greater than in any previous year on record [1,2]. U.S. children are 11 times more likely to die of a firearm injury than children in other high-income countries [3], and firearm injury is the leading cause of death for Americans age 1–19 (largely due to homicide) [4]. This epidemic also has a disproportionate impact on people who are Black, Indigenous and/or people of color (BIPOC), especially in socially vulnerable, underserved communities. Firearm homicide is the leading cause of death for Black males 15–34 years old, and Black men are 10–20 times more likely to be killed by a gun than White men [1]. American Indian and Alaskan Native men are 3–4 times more likely to die by firearm suicide than White men [2].

Despite the growing frequency of school shootings and public mass shootings in the U.S, mass shootings in schools and other public spaces make up a minority of U.S. firearm injuries and deaths [1]. Instead, most U.S. firearm deaths are homicide and suicide. Indeed, firearms are the most common mechanism by which U.S. adults and children die from suicide, with approximately 53% of suicide deaths resulting from firearms in 2021 [1]. Firearms are also the most common mechanism of homicide, causing 80% of U.S. homicide deaths in 2021 [1]. Intimate partner homicide is most frequently completed with firearms in the U.S. [2]. Most non-fatal firearm injuries are due to assault [1].

The impact of these violent injuries on individuals, families, communities and U.S. society is massive [5], ranging from anxiety, to substance use, to increased firearm purchasing. Studies suggest a strong, but not complete, correlation between rates of firearm ownership and number of firearm deaths [6]. Other causes of injury and death are also at play. For example, underinvestment in the physical structure of a city has been shown to influence rates of firearm ownership and death. Firearm ownership practices related to beliefs around the need for self-protection may contribute to unintentional injury rates. A history of firearm trauma–and resultant patterns of post-traumatic stress, substance use, involvement in the criminal justice system, and carrying firearms for self-protection–may lead to perpetuating cycles of firearm injury and death [7]. Structural racism, which includes residential segregation, low-quality schools, limited healthcare resources, and lack of implementation of effective criminal justice policy, further contributes to adverse social determinants of health that increase risk for firearm assault [8].

Firearm injuries—regardless of intent—are influenced by multiple levels of the social-ecologic model, which makes legislation in isolated states or regions a challenging mode of intervention when used in isolation [5]. Unfortunately, since the mid-1990s, federal funding and legislation addressing firearm injury as a public health problem has lagged far behind the burden of this disease [9]. In parallel, the U.S. has become a global leader in population adjusted rates of firearm ownership (access) [10]. Approximately 400 million firearms are owned by U.S. private citizens [6], and millions of Americans became first-time firearm owners during the COVID-19 pandemic.

Yet all hope is not lost.

The field of public health is well positioned to contribute many actionable solutions that are ready to be deployed and scaled. Solutions to any public health epidemic must be multi-factorial, including economic, structural, legislative, and educational interventions–and solutions to the problem of firearm violence are urgently needed.

Recent systematic reviews have evaluated broad classes of gun legislative polices that have been implemented in some U.S. states [11,12]. Policies correlated with reduced suicide, violent crime and homicide rates include minimum age requirements, prohibitions associated with domestic violence, surrender of firearms by prohibited possessors, background checks, and waiting periods. Child access prevention laws—also known as safe storage laws—appear to be among the most effective. Such laws, which require guns to be stored locked, unloaded, and kept apart from ammunition, correlate with reduced suicides and homicides among young people [12]. Extreme risk protection orders (“red flag laws”) are also promising tools to reduce firearm suicide and mass shootings. However, the current U.S. political environment and the recent Supreme Court *Bruen* decision limit both legislation and its enforcement, on both the state and federal level. Moreover, the history of public health teaches us that legislation alone rarely solves a public health problem.

A promising public health concept is that of harm reduction. Harm reduction is a comprehensive prevention strategy that emphasizes engaging directly with people at risk for harm. This strategy offers low-threshold options to access prevention and treatment that facilitate individual autonomy, and improve physical and mental health and social wellbeing [13]. Harm reduction includes several core principles (Table 1).

**Table 1:** Core principles of harm reduction in the public health context.

Principle	Definition	Example of harm reduction-based strategy for firearm injury prevention
Humanism	We value, care for, respect, and dignify people as individuals. Understanding why people make decisions empowers change.	Collaboration with firearm owners is necessary to respect and dignify choices that may reduce (but not eliminate) risk of injury.
Pragmatism	None of us will ever achieve perfect health behaviors. Health behaviors and the ability to change them are influenced by social and community norms (i.e., behaviors do not occur within a vacuum).	Reductions in injury patterns will be enhanced by addressing the structural- and community-level drivers of injury.
Individualism	Every person presents with their own needs and strengths. People require a spectrum of intervention options.	Hospital-based violence intervention programs can provide social- and case-based services through individualized treatment plans.
Incrementalism	Any positive change is a step toward improved health, and positive change can take years. It is important to understand and plan for backward movements.	Safer storage of firearms lies along a spectrum from storing all firearms locked and unloaded, to storing all firearms unlocked and loaded.

Firearm injury prevention interventions well-aligned with the principles of harm reduction exist. For example, individual-level interventions such as counseling on access to lethal means (CALM) and anticipatory guidance on safe storage of firearms have the potential to decrease firearm deaths from both suicide and accidental injury [14]. Similarly, the American Academy of Pediatrics recommends “anticipatory guidance” around firearm storage, in which health care providers discuss safe firearm storage with parents of children and teens–similar to how they discuss bicycle helmets and access to medicines–to limit unauthorized access.

Another actionable, non-legislative, harm-reduction-centered approach to firearm injury prevention includes violence intervention or interruption programs, including hospital-based violence intervention programs (HVIPs), that address individual root causes of community-based firearm assault. Grounded in a peer support and case management model, these programs engage violent injury survivors in prevention efforts aimed at improving health and safety of patients and their families after hospital-based care for violent injuries. Used in concert with broad multidisciplinary strategies, HVIPs may contribute to lower reinjury rates after one year of program participation and mitigate the secondary effects of violence (e.g. post-traumatic stress disorder, long-term disability, missed school) [5,15].

Finally, collective prevention efforts are needed to increase community engagement and normative change. Several regional and national organizations are working towards this goal of equitable approaches and solutions that mitigate the undue burden of firearm violence, especially among our most vulnerable populations. For example, the Health Alliance for Violence Intervention builds and connects violence intervention programs and promotes equity for victims of violence globally. The community-based public safety (CBPS) collective is a recently emerged group of firearm violence prevention workers building capacity for research and advocacy to address community violence in Black and Brown communities by strengthening neighborhood leadership through investment in the education, advocacy and training of community-based public safety practitioners and organizations. The Penn Urban Health Lab has worked with local government and community organizations to understand the ways in which the physical attributes of where people, live, work, and play influence mental health, substance use, and violent crime. Through partnerships with 4-H, research is being conducted to evaluate the efficacy of bystander intervention curricula for youth in shooting sports programs. Hospitals (e.g., Northwell Health), health systems (e.g. Kaiser Permanente), and academic centers (e.g., University of Michigan) are investing in comprehensive firearm injury prevention collaborations and research.

There is great potential to successfully disrupt this U.S. crisis of firearm injury and death. To attain this goal, however, substantial intentional investment in research, programming, personnel, policies, and community infrastructure is needed. We remind readers, however, that public health professionals, health care providers, and community members can make a difference, regardless of politics. We can collect data, implement evidence-based programs, advocate for evidence-based policies and regulation, and work to shift culture and reduce harm—today.

## References

[1] Web-based Injury Statistics Query and Reporting System (WISQARS) [online] Leading Cause of Death Reports, 1981–2020 [Internet]. Centers for Disease Control and Prevention, National Center for Injury Prevention and Control. 2023 [cited February 18, 2023]. Available from: https://www.cdc.gov/injury/wisqars/fatal.html.

[2] CDC Wonder: U.S. Department of Health and Human Services; [updated February 1, 2023. Available from: https://wonder.cdc.gov/.

[3] Richardson EG, Hemenway D. Homicide, suicide, and unintentional firearm fatality: comparing the United States with other high-income countries, 2003. The Journal of trauma. 2011;70(1):238–43. doi: 10.1097/TA.0b013e3181dbaddf 20571454

[4] Rees CA, Monuteaux MC, Steidley I, Mannix R, Lee LK, Barrett JT, et al. Trends and Disparities in Firearm Fatalities in the United States, 1990–2021. JAMA network open. 2022;5(11):e2244221. doi: 10.1001/jamanetworkopen.2022.44221 36445703 PMC9709653

[5] Centers for Disease Control and Prevention National Center for Injury Prevention and Control Division of Violence Prevention. The Social-Ecologic Model: A Framework for Prevention 2021 [Available from: https://www.cdc.gov/violenceprevention/about/social-ecologicalmodel.html.

[6] Kaufman EJ, Morrison CN, Branas CC, Wiebe DJ. State Firearm Laws and Interstate Firearm Deaths From Homicide and Suicide in the United States: A Cross-sectional Analysis of Data by County. JAMA internal medicine. 2018;178(5):692–700. doi: 10.1001/jamainternmed.2018.0190 29507953 PMC5885268

[7] Richardson JB, St Vil C, Sharpe T, Wagner M, Cooper C. Risk factors for recurrent violent injury among black men. The Journal of surgical research. 2016;204(1):261–6. doi: 10.1016/j.jss.2016.04.027 27451895

[8] Bailey ZD, Krieger N, Agénor M, Graves J, Linos N, Bassett MT. Structural racism and health inequities in the USA: evidence and interventions. Lancet (London, England). 2017;389(10077):1453–63. doi: 10.1016/S0140-6736(17)30569-X 28402827

[9] Stark DE, Shah NH. Funding and Publication of Research on Gun Violence and Other Leading Causes of Death. JAMA. 2017;317(1):84–5. doi: 10.1001/jama.2016.16215 28030692

[10] Naghavi M, Marczak LB, Kutz M, Shackelford KA, Arora M, Miller-Petrie M, et al. Global Mortality From Firearms, 1990–2016. Jama. 2018;320(8):792–814. doi: 10.1001/jama.2018.10060 30167700 PMC6143020

[11] Gun Policy Research Review: © 1994–2023 RAND Corporation; [updated January 10, 2023. Available from: https://www.rand.org/research/gun-policy/analysis.html.

[12] Gammon K. Laws that keep kids away from guns reduce deaths, whereas others lead to more shootings: Science; 2023 [updated January 10, 2023. Available from: https://www.science.org/content/article/u-s-gun-research-report-child-access-prevention-laws-cut-firearm-deaths#:~:text=The%20researchers%20found%20evidence,and%20homicides%20among%20young%20people.

[13] Harm Reduction 5600 Fishers Lane, Rockville, MD 20857: Substance Abuse and Mental Health Services Administration; [updated August 16, 2022. Available from: https://www.samhsa.gov/find-help/harm-reduction.

[14] McCourt AD. Firearm Access and Suicide: Lethal Means Counseling and Safe Storage Education in a Comprehensive Prevention Strategy. American journal of public health. 2021;111(2):185–7. doi: 10.2105/AJPH.2020.306059 33439717 PMC7811084

[15] Affinati S, Patton D, Hansen L, Ranney M, Christmas AB, Violano P, et al. Hospital-based violence intervention programs targeting adult populations: an Eastern Association for the Surgery of Trauma evidence-based review. Trauma surgery & acute care open. 2016;1(1):e000024. doi: 10.1136/tsaco-2016-000024 29766064 PMC5891700

